# Effect of Air Quality on the Risk of Emergency Room Visits in Patients With Atrial Fibrillation

**DOI:** 10.3389/fcvm.2021.672745

**Published:** 2021-05-11

**Authors:** Bin Liang, Xiaonan He, Xin Du, Xiaoxia Liu, Changsheng Ma

**Affiliations:** ^1^Department of Cardiology, Beijing Anzhen Hospital, Capital Medical University, Beijing, China; ^2^Department of Cardiology, The Second Hospital of Shanxi Medical University, Taiyuan, China; ^3^Emergency Critical Care Center, Beijing Anzhen Hospital, Capital Medical University, Beijing, China

**Keywords:** PM2.5, atrial fibrillation, risk of emergency room visit, monsoon climate region, patients

## Abstract

**Background:** We investigated the effect of particulate matter with aerodynamic diameter <2.5 μm (PM2.5) and meteorological conditions on the risk of emergency room visits in patients with atrial fibrillation (AF) in Beijing, which is considered as a monsoon climate region.

**Methods:** In this case-crossover design study, medical records from patients with AF who visited the Critical Care Center in the Emergency Department of Anzhen Hospital from January 2011 through December 2014 and air quality and meteorological data of Beijing during the same period were collected and analyzed using Cox regression and time-series autocorrelation analyses.

**Results:** A total of 8,241 patients were included. When the average PM2.5 concentration was >430 μg/m^3^, the risk of emergency room visits for patients with uncomplicated AF, AF combined with cardiac insufficiency, and AF combined with rheumatic heart disease increased by 12, 12, and 40%, respectively. When the average PM2.5 concentration was >420 μg/m^3^, patients with AF combined with diabetes mellitus had a 75% increased risk of emergency room visits, which was the largest increase in risk among all types of patients with AF. When the average PM2.5 concentration was >390 μg/m^3^, patients with AF combined with acute coronary syndrome had an approximately 30% increased risk of emergency room visits, which was the highest and fastest increase in risk among all types of patients with AF. The risk of emergency room visits for patients with AF was positively correlated with air quality as the time lag proceeded, with an autocorrelation coefficient of 0.223 between the risk of emergency room visits and air quality in patients with AF on day 6 of the time lag.

**Conclusion:** Exposure to certain concentrations of PM2.5 in a monsoon climate region significantly increased the risk of emergency room visits in patients with AF.

## Introduction

With the advancement in industrialization, air pollution is on the rise. The increase in the concentration of various pollutants, such as PM2.5, has been closely associated with various diseases, including lung, cardiovascular, kidney, and eye diseases, according to an increasing number of studies (1–4). A joint opinion from the World Heart Federation, American College of Cardiology, American Heart Association, and the European Society of Cardiology has been published against air pollution recently (5).

Epidemiological studies have shown that air pollution is associated with the development of cardiovascular disease (CVD) (6, 7). A study on the link between CVD and air pollution particulate matter found that a 10 μg/m^3^ increase in PM concentration was associated with a 4% and 10% increase in the incidence of total CVD and ischemic heart disease, respectively (8). In addition, the number of CVD hospitalizations increased with increases in air pollution index (9). These studies suggested that exposure to a certain concentration of PM2.5 may significantly increase morbidity, hospitalization rates, and mortality rates of patients with CVD and that reducing PM2.5 exposure may improve cardiovascular event outcomes (10).

AF is the most common arrhythmia observed in clinical practice, with a complex etiology and an incidence that increases sharply with age (11, 12). Recent studies have shown that PM2.5 exposure may contribute to arrhythmogenesis (13). Additional studies have found that PM2.5 exposure is associated with increased risk of incident AF (14). Possible reasons include (i) the adverse effects of PM2.5 on cardiac autonomic regulation (15–17); (ii) possible inflammation, oxidative stress, and altered atrial pressure caused by PM2.5 (18–20); and (iii) other unidentified causes.

However, only a few studies have focused on the synergistic effects of air pollution and meteorological parameters on AF morbidity. Therefore, in this study, we analyzed the effect of meteorological conditions and PM2.5 exposure on the risk of emergency room visits in patients with AF in a monsoon climate region to further identify risk factors for AF and to provide a theoretical basis on the importance of air quality improvement.

## Materials and Methods

### Data Related to PM2.5

Beijing is located in northern China, which is a monsoon climate region with four distinct seasons including cold and windy dry winters and hot and humid summers. In this study, four air quality monitoring stations within an average distance of 7.5 km from Beijing Anzhen Hospital were selected, and data on the 24-h average PM2.5 concentration from January 1, 2011 to December 31, 2014 were retrieved from the website of the Ministry of Ecology and Environment of the People's Republic of China and used as a representative air quality measurement for each day (http://www.mee.gov.cn/).

### Data Related to Meteorological Conditions

The Beijing Regional Climate Center is an accurate and reliable electronic weather database. We collected data related to meteorological conditions from January 1, 2011 to December 31, 2014 from the measurement points of the 16 districts of Beijing through the website (http://bj.cma.gov.cn/). The main data included average temperature (°C), minimum temperature (°C), maximum temperature (°C), diurnal temperature range (DTR, °C), relative humidity (%), average wind speed, and air pressure (hPa). DTR refers to the difference between the maximum and minimum temperatures in 1 day.

### Data Related to Clinical Information

Medical records from all patients with AF who visited the Critical Care Center in the Emergency Department of Anzhen Hospital from January 1, 2011 to December 31, 2014, were collected, including sex, age, comorbidities (diabetes, acute coronary syndrome, rheumatic heart disease, and cardiac insufficiency), and the time of emergency room visits. Private information, such as name and ID number, was not collected. The inclusion criteria for the medical study were residence in Beijing and its vicinity and a diagnosis of AF based on the International Statistical Classification of Diseases and Related Health Problems, 9^th^ edition, confirmed by electrocardiography. Patients with atrial fibrillation caused by cor pulmonale, hyperthyroidism and traffic accidents were excluded.

### Quality Control

Medical records were obtained from a tertiary referral center and uploaded after review to ensure accuracy. The PM2.5 concentration and meteorological conditions data were obtained from the release of the Ministry of Environmental Protection and the China Meteorological Administration, which are authoritative standards in this regard.

### Statistical Methods

SPSS version 25.0 was used for statistical analysis. Normally distributed measures are expressed as mean ± standard deviation (SD), and discrete variables are described with median and interquartile range. In order to clarify the close degree and direction of the correlation between air quality and meteorological conditions, Pearson correlation was used to analyze the correlation between them. The daily frequency of atrial fibrillation was simulated by stepwise Cox regression analysis model to study the relationship between daily atrial fibrillation attacks and air quality PM2.5 concentration in patients with atrial fibrillation without other diseases, cardiac insufficiency, rheumatic heart disease, diabetes and acute coronary syndrome. The lagged correlation between air quality and AF episodes was investigated by autocorrelation analysis of the time series. *P* < 0.05 was considered as a statistically significant difference.

## Results

### Characteristics of the Climate in Beijing

**Figure 1:**
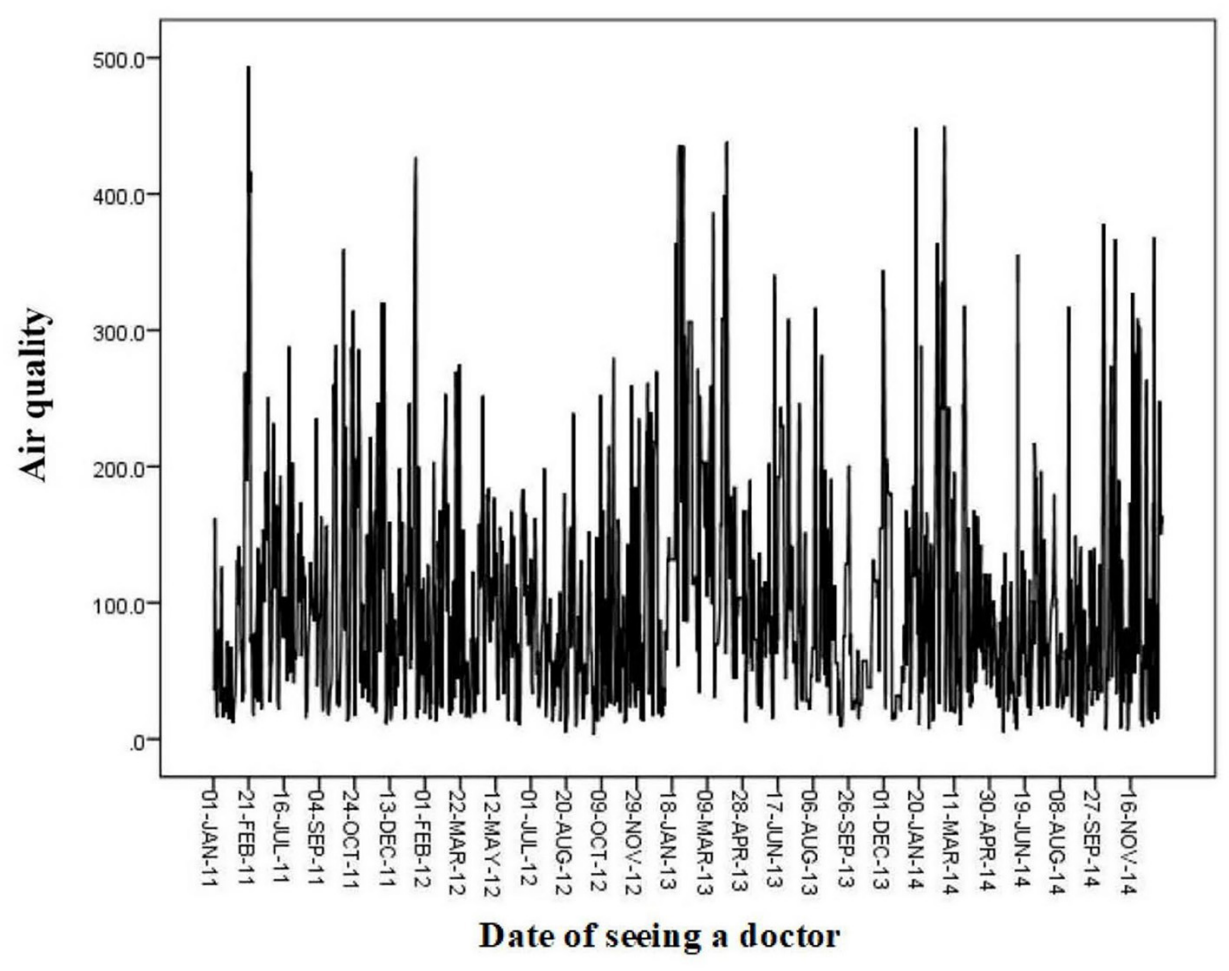
Daily air quality, 2011–2014.

Beijing has four distinct seasons and a typical northern temperate and semi-humid continental monsoon climate. During 2011–2014, the annual average temperature in Beijing was 12.41–12.65°C, with the highest annual average temperature between 17.55°C and 17.81°C, the lowest annual average temperature between 7.73°C and 7.79°C, and the average PM2.5 concentration between 105.02 and 106.90 μg/m^3^ (Table 1). Air quality was poor(PM2.5 concentration was >35 μg/m^3^) in February and October 2011; February 2012; January, March, June, August, and December 2013; and January, March, June, August, September, and November 2014 (Figure 1). Pearson's correlation analysis, which was used to examine the correlation between air quality and meteorological conditions, showed that air quality was negatively correlated with average air temperature, maximum air temperature, minimum air temperature, diurnal temperature difference, average wind speed, and average daily surface temperature, and positively correlated with relative humidity (Table 2).

**Table 1:** The information of air quality and meteorological conditions in Beijing, 2011–2014.

	**Minimum**	**P25**	**Median**	**Mean (SD)**	**P75**	**Maximum**
Average temperature (°C)	−10	1.30	13.60	12.53 (0.12)	23.30	32
Maximum temperature (°C)	−6	6.50	19.90	17.68 (0.13)	27.90	32
Minimum temperature (°C)	−14	−2.70	7.90	7.85 (0.12)	18.25	28
DTR (°C)	2	7.10	9.30	9.69	12.10	21
Relative humidity (%rh)	0	38	54	53.45 (0.22)	69.00	97
Average wind speed (m/s)	1	1.50	1.90	2.09 (0.01)	2.50	7
Average daily surface temperature (°C)	−12	0.20	14.20	13.36 (0.14)	25.10	37
Air pressure (kPa)	9,900	10,049	10,133	10,131.87 (1.09)	10,218	10,392
Air quality (μg/m^3^)	3	42.42	84.04	105.96 (0.94)	146.38	552.50

*Minimum refers to the minimum value; P25 refers to the 25th percentile; median refers to the median; P75 refers to the 75th percentile, and maximum refers to the maximum value. DTR:diurnal temperature range*.

**Table 2:** Pearson's correlation analysis of air quality and meteorological conditions.

	**Average temperature**	**Maximum temperature**	**Minimum temperature**	**DTR**	**Relative humidity**	**Average wind speed**	**Average daily surface temperature**	**Air pressure**	**Air quality**
Average temperature	1	0.988^**^	0.982^**^	0.123	0.317^**^	−0.009	0.988^**^	−0.870^**^	−0.143^**^
Maximum temperature	0.988^**^	1	0.949^**^	0.255	0.262^**^	−0.018	0.980^**^	−0.866^**^	−0.151^**^
Minimum temperature	0.982^**^	0.949^**^	1	−0.051	0.405^**^	−0.008	0.964^**^	−0.853^**^	−0.112^**^
DTR	0.123	0.255	−0.051	1	−0.429	−0.023	0.153	−0.127	−0.115^**^
Relative humidity	0.317^**^	0.262^**^	0.405^**^	−0.429	1	−0.452^**^	0.283^**^	−0.347^**^	0.366^**^
Average wind speed	−0.009	−0.018	−0.008	−0.023	−0.452^**^	1	0.024^*^	−0.012	−0.260^**^
Average daily surface temperature	0.988^**^	0.980^**^	0.964^**^	0.153	0.283^**^	0.024^*^	1	−0.860^**^	−0.144^**^
Air pressure	−0.870^**^	−0.866^**^	−0.853^**^	−0.127	−0.347^**^	−0.012	−0.860^**^	1	0.015
Air quality	−0.143^**^	−0.151^**^	−0.112^**^	−0.115^**^	0.366^**^	−0.260^**^	−0.144^**^	0.015	1

* *P < 0.05 (two-tailed) with significant correlation*;

** *P < 0.01 (two-tailed) with significant correlation*.

### Characteristics of Patients With AF

**Figure 2:**
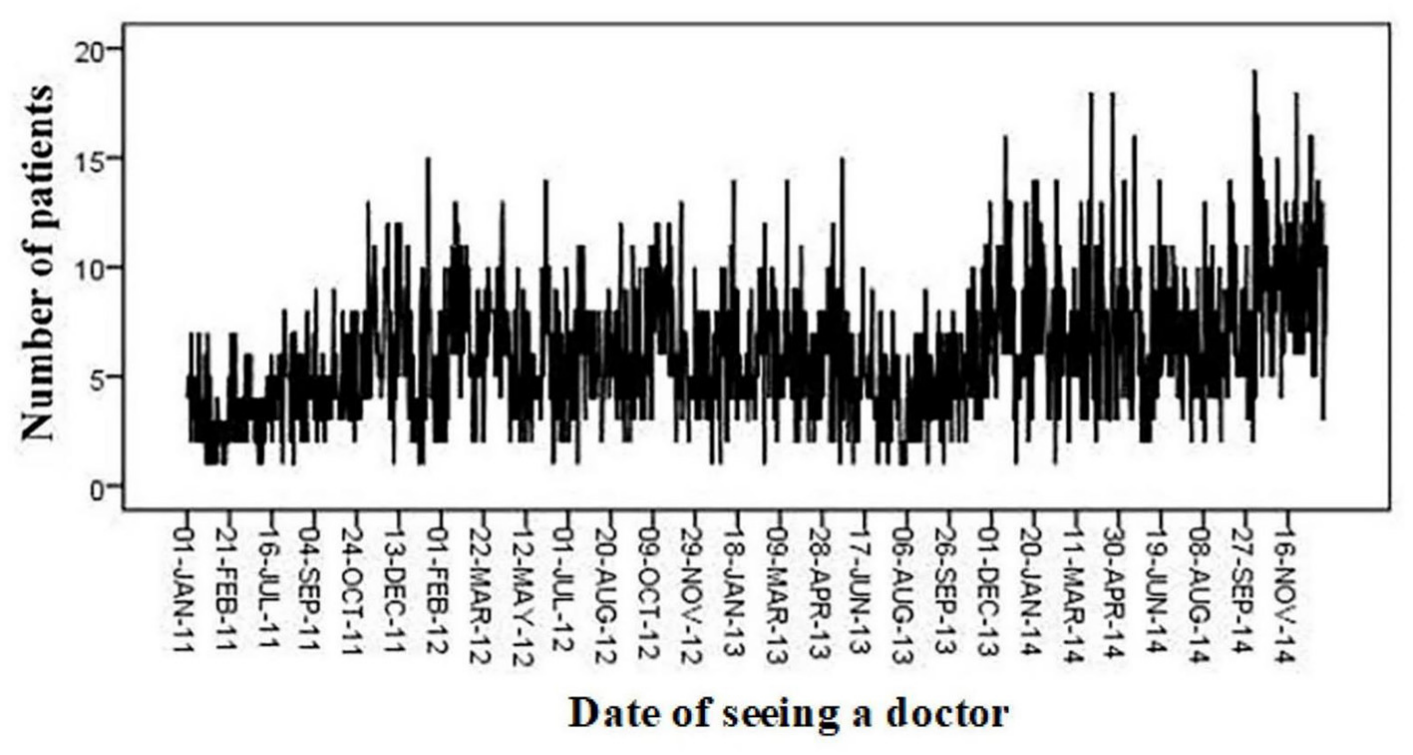
Number of patients with atrial fibrillation seen in emergency departments per day, 2011–2014.

From 2011 to 2014, 8,241 patients with AF in Beijing and its vicinity were treated in the Critical Care Center at the Emergency Department of Anzhen Hospital. The mean age of all patients with AF observed in the Emergency Department was 60 years, and the mean age of patients with uncomplicated AF and AF combined with diabetes, acute coronary syndrome, rheumatic heart disease, or cardiac insufficiency was 60.08, 60.85, 57.37, 56.43, and 64.44 years, respectively (Table 3). More patients with AF visited the Emergency Department in October and December 2011; February and June 2012; January, March, June, and December 2013; and January, March, April, June, September, and November 2014 (Figure 2), which was generally consistent with the months with poor air quality, as shown in Figure 1, suggesting that poor air quality may have led to an increase in the number of emergency visits for patients with AF.

**Table 3:** Statistical description of the general information of patients with atrial fibrillation visiting the Emergency Department in 2011–2014.

	**Minimum (years)**	**P25 (years)**	**Median (years)**	**Mean (SD) (years)**	**P75 (years)**	**Maximum (years)**
Age	18	51	60	60 (14.37)	71	98
Atrial fibrillation	18	51	60	60.08 (0.16)	71	98
Atrial fibrillation combined with diabetes mellitus	21	53	61	60.85 (0.53)	73	96
Atrial fibrillation combined with acute coronary syndrome	26	54	58	57.37 (0.69)	63	88
Atrial fibrillation combined with rheumatic heart disease	18	47	49	56.43 (0.57)	67	86
Atrial fibrillation combined with cardiac insufficiency	26	56	71	64.44 (1.12)	72	87

### Impact of PM2.5 on the Risk of Emergency Room Visits for Patients With AF

**Figure 3:**
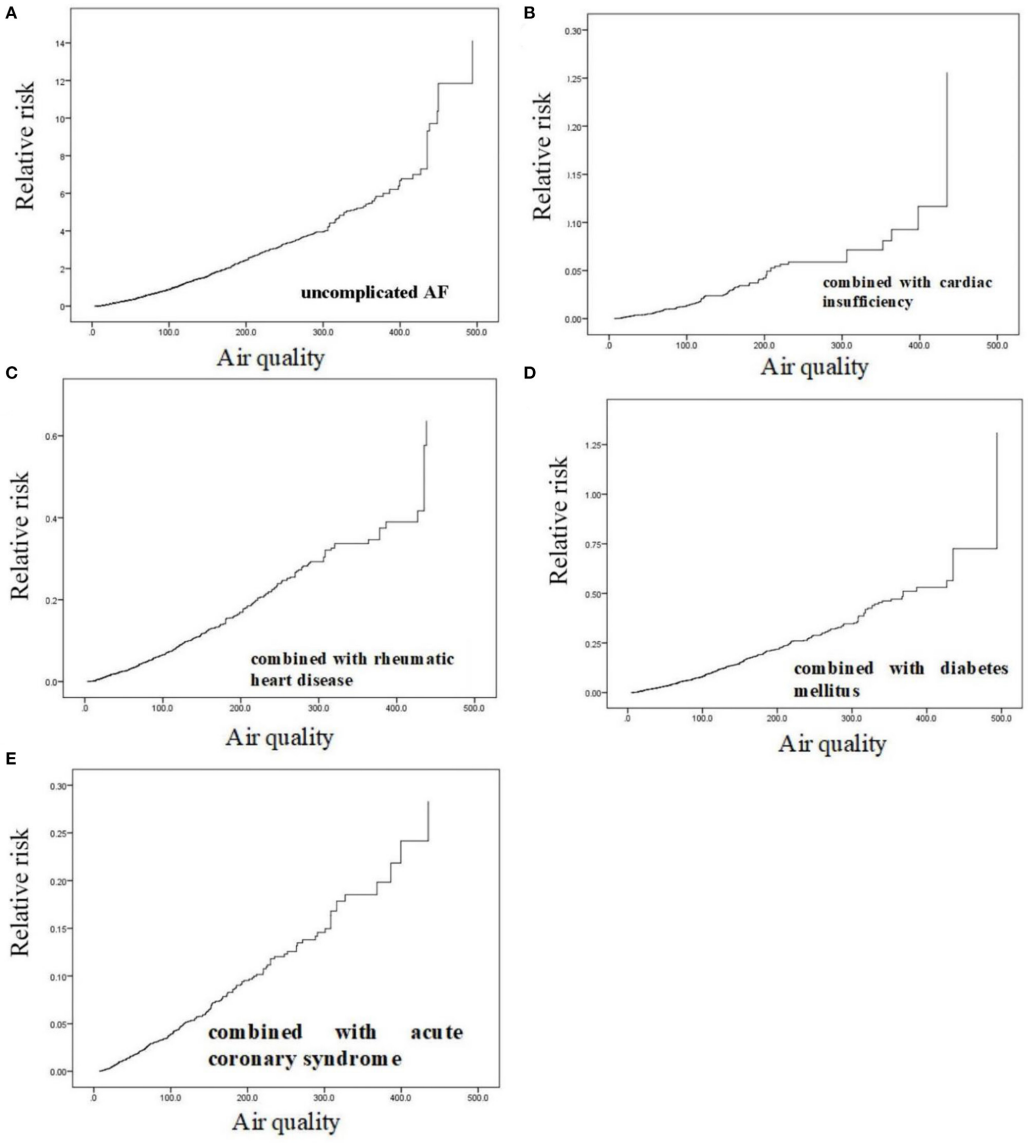
Correlation analysis of particulate matter with aerodynamic diameter <2.5 μm and risk of emergency room visits for patients with atrial fibrillation. **(A)** uncomplicated AF; **(B)** AF combined with cardiac insufficiency; **(C)** AF combined with rheumatic heart disease; **(D)** AF combined with diabetes mellitus; **(E)** AF combined with acute coronary syndrome.

Cox regression analysis showed that air pollution increased the risk of emergency room visits for patients with AF. When the average PM2.5 concentration was >430 μg/m^3^, the risk of emergency room visits for patients with uncomplicated AF, AF combined with cardiac insufficiency, and AF combined with rheumatic heart disease increased by 12, 12, and 40%, respectively (Figures 3A–C). When the average PM2.5 concentration was >420 μg/m^3^, patients with AF combined with diabetes mellitus had a 75% increased risk of emergency room visits, which was the largest increase in risk among all types of patients with AF (Figure 3D). When the average PM2.5 concentration was >390 μg/m^3^, patients with AF combined with acute coronary syndrome had an ~30% increased risk of emergency room visits, which was the fastest increase in risk among all types of patients with AF (Figure 3E).

### Autocorrelation Analysis of Air Quality and Time Series Data of Emergency Room Visits for AF

The effect of short-term PM2.5 exposure on the risk of emergency room visits for patients with AF was investigated by autocorrelation analysis of the time series data. The results showed that air quality was positively associated with the risk of emergency room visits for patients with AF with a time lag, and the autocorrelation coefficient for air quality and the risk of emergency room visits for AF was 0.223 on day 6 of the time lag (Table 4).

**Table 4:** Autocorrelation analysis of the time series of the air quality and risk of emergency room visits for patients with atrial fibrillation.

**Number of days lagged**	**Autocorrelation**	**Standard error**	**Value**	**Significance**
1	−0.467	0.027	292.814	0.000
2	−0.028	0.027	293.899	0.000
3	−0.044	0.027	296.449	0.000
4	0.101	0.027	310.117	0.000
5	−0.046	0.027	312.920	0.000
6	0.223	0.027	380.078	0.000

## Discussion

A study published in the *Lancet* found that air pollution contributed to CVD morbidity and premature human death (21). The American Heart Association indicated that there was a clear causal relationship between air pollutants and CVD morbidity. The World Health Organization reported that air pollution was strongly associated with CVD mortality. There is also clear research evidence in China that air pollutant PM2.5 affected the clinical outcomes of CVD (22).

Short-term air pollution exposure may lead to a variety of cardiac arrhythmias, including AF. Several studies suggested that air pollutants may cause cardiac autonomic dysregulation, which may induce atrial electrophysiological changes (23–26). In addition, inflammation and oxidative stress may also cause changes in atrial pressure, leading to episodes of AF.

There is a lack of epidemiological evidence on the effects of air pollution on the incidence, recurrence, and clinical outcomes of AF. To date, only two studies have investigated the association between exposure to air pollution and the incidence of AF. One of these studies examined the association between AF and nitrogen dioxide, whereas the other study examined the relationship between PM2.5 and PM10 and AF (27). These studies suggested an association between air pollution and AF. Consistent with this, we found that the risk of AF episodes increased with poorer air quality, which may be associated with higher PM2.5 concentrations. The risk of AF episodes increased significantly when PM2.5 was >390 μg/m^3^, and the risk of emergency room visits increased abruptly, which was consistent with the results of previous studies.

Several time-series analyses have examined the short-term effects of air pollution on cardiovascular events and found that exposure to air pollution (especially PM2.5 and NO_2_) increased the risk of AF (28, 29). In this study, we found a significant positive association between PM2.5 concentration and the risk of emergency room visits in patients with AF by time-series autocorrelation analysis. This result suggested that short- to medium-term exposure to certain PM2.5 concentrations was significantly associated with the risk of emergency room visits for patients with AF. This finding is consistent with the evidence of the arrhythmogenic effects of PM2.5 mentioned above.

In previous epidemiological studies examining the correlation between short-term air pollution exposure and AF, mainly hospitalized patients or individuals with continuous rhythm monitoring (i.e., patients with implanted devices or individuals undergoing ambulatory electrocardiographic monitoring) were included (30). Most studies examined patients with implantable cardioverter defibrillators (ICDs) and revealed an association between PM and non-allergic diseases or episodes of ventricular fibrillation (31). However, because heart failure is one of the most common causes for the implantation of an ICD, the clinical characteristics of patients with ICDs are not representative of those of the general population; therefore, the finding that PM concentration had an effect on the incidence of AF in patients with ICDs in a high-risk susceptible population cannot be extrapolated to the general population. In this study, data from all patients with AF visiting the Emergency Department of Anzhen Hospital during the study period were collected, and the results of the study were more relevant to the actual clinical level.

This study is of great clinical significance; however, it also has limitations. First, information was collected through the Anzhen Hospital patient record database, where only patient records with a clear AF diagnosis by a physician could be obtained, which may have led to missing of undiagnosed cases. Second, the date of AF diagnosis was used, which may not be the exact date of AF onset. Third, considering the spontaneous termination or intervention, the true incidence of paroxysmal AF may have been underestimated. Finally, this study used data related to air pollution only at the patients' place of residence and lacked information on exposure to pollutants in other places, including exposure in occupational settings or while commuting, as well as indoor pollutants; thus, we could not accurately characterize the cumulative exposure to environmental and indoor air pollution. Our next steps will be to thoroughly examine potential susceptible subgroups, understand the impact of environmental exposures on CVDs, and gain a deeper understanding of the overall human cost of economic development by finding the true time of AF onset and collecting information on other air pollution exposures in patients.

Exposure to certain concentrations of PM2.5 significantly increased the risk of emergency room visits in patients with AF. There was a correlation between short- and medium-term exposure to the air pollutant PM2.5 and emergency room visits in patients with AF. These findings highlight the importance of air quality improvement and provide a rationale for developing interventions to reduce CVD risk in the population.

## Data Availability Statement

The raw data supporting the conclusions of this article will be made available by the authors, without undue reservation.

## Author Contributions

BL and XH: study concept, design, analysis, interpretation of data, and drafting of the article. BL and XL: drafting of the article. XD and CM: critical revision of the manuscript for important intellectual content. All authors contributed to the article and approved the submitted version.

## Conflict of Interest

The authors declare that the research was conducted in the absence of any commercial or financial relationships that could be construed as a potential conflict of interest.
